# The burden of serious health-related suffering among cancer decedents: Global projections study to 2060

**DOI:** 10.1177/0269216320957561

**Published:** 2020-09-18

**Authors:** Katherine E Sleeman, Barbara Gomes, Maja de Brito, Omar Shamieh, Richard Harding

**Affiliations:** 1 King’s College London, Faculty of Nursing, Midwifery and Palliative Care, Cicely Saunders Institute, London, UK; 2 Faculty of Medicine, University of Coimbra, Coimbra, Portugal; 3 Department of Palliative Care, King Hussein Cancer Center, Amman, Jordan

**Keywords:** Terminal care, palliative care, pain, cancer, stress, psychological

## Abstract

**Background:**

Palliative care improves outcomes for people with cancer, but in many countries access remains poor. Understanding future needs is essential for effective health system planning in response to global policy.

**Aim:**

To project the burden of serious health-related suffering associated with death from cancer to 2060 by age, gender, cancer type and World Bank income region.

**Design:**

Population-based projections study. Global projections of palliative care need were derived by combining World Health Organization cancer mortality projections (2016–2060) with estimates of serious health-related suffering among cancer decedents.

**Results:**

By 2060, serious health-related suffering will be experienced by 16.3 million people dying with cancer each year (compared to 7.8 million in 2016). Serious health-related suffering among cancer decedents will increase more quickly in low income countries (407% increase 2016–2060) compared to lower-middle, upper-middle and high income countries (168%, 96% and 39% increase 2016-2060, respectively). By 2060, 67% of people who die with cancer and experience serious health-related suffering will be over 70 years old, compared to 47% in 2016. In high and upper-middle income countries, lung cancer will be the single greatest contributor to the burden of serious health-related suffering among cancer decedents. In low and lower-middle income countries, breast cancer will be the single greatest contributor.

**Conclusions:**

Many people with cancer will die with unnecessary suffering unless there is expansion of palliative care integration into cancer programmes. Failure to do this will be damaging for the individuals affected and the health systems within which they are treated.

**What is already known about the topic**

- Projections studies estimate that by 2060, almost 50 million people will die each year with serious health-related suffering, and that cancer is the biggest single contributor to the increase in suffering.
- Different cancer sub-types are associated with varying symptom profiles and patterns of palliative care need. Understanding how different cancer sub-types contribute to the projected increase in serious health-related suffering would enable more effective health system planning, but has not been described.

**What this paper adds**

- Serious health-related suffering associated with death from cancer will double over the next four decades; by 2060 there will be 16.3 million people dying with cancer each year who experience serious health-related suffering; serious health-related suffering among cancer decedents is increasing most rapidly in low income countries, and in people aged over 70.
- The cancer types associated with the greatest burden of serious health-related suffering are lung cancer in higher income countries, and breast cancer in lower income countries.

**Implications for policy**

- Palliative care must be integrated into cancer control programmes. This is particularly important in low income countries where needs are increasing rapidly and current access is poor.

## Background

Cancer has been described as a global problem that lacks a global solution.^1^ It is estimated that in 2017 there were 24.5 million incident cancer cases globally and 9.6 million deaths from cancer.^1^ The Lancet Commission on Palliative Care and Pain Relief estimated that 90% of people who die with cancer globally experience serious health related suffering and would benefit from palliative care.^2,3^ Palliative care improves symptom control and quality of life for patients and their families.^4^ In addition, its provision can strengthen health systems through avoidance of catastrophic costs, particularly in low and middle income countries.^5^ For these reasons, the American Society of Clinical Oncology recommends early integration of palliative care in the care pathway for all patients with metastatic cancer and/or high disease burden, including in resource constrained settings.^6^ Universal Health Coverage goals call on governments to ensure that “all people and communities can use the . . . palliative health services they need’.^7^

We have recently shown that global palliative care needs among decedents will double over the next four decades, and that cancer is the single largest contributor to this projected need.^3^ However, this analysis did not explore differences by cancer sub-type, or examine the age and gender distribution of serious health-related suffering among cancer decedents in different world regions, which is essential for effective health system planning. The aim of the projections we hereby present, was to estimate the burden of serious health-related suffering associated with death from cancer to 2060 by age, gender, cancer type and world region.

## Method

This was a population-based projections study. Projections of palliative care need for decedents with cancer were derived by combining World Health Organization (WHO) projections for cancer mortality (2016–2060) with estimates of palliative care need using methods described previously.^3^ In short, we extracted information on mortality projections for malignant neoplasms and leukaemia for 2016 to 2060 from the WHO.^8^ These projections estimate future mortality by cause of death, age, gender and geographical region. We used methods developed by the Lancet Commission on Palliative Care and Pain Relief to estimate the proportion of decedents with cancer who are likely to have palliative care needs.^2^ The Lancet Commission developed a measure of serious health-related suffering based on the prevalence of physical and psychological symptom prevalence in twenty different conditions. For both malignant neoplasms and leukaemia, the Lancet Commission estimated that 90% of decedents would experience serious health-related suffering. For this analysis, we therefore applied the multiplier 0.9 to the WHO mortality projections to project palliative care needs among cancer decedents, and these were described by age, gender and cancer type, and stratified by World Bank income region.

## Results

**Figure 1.**
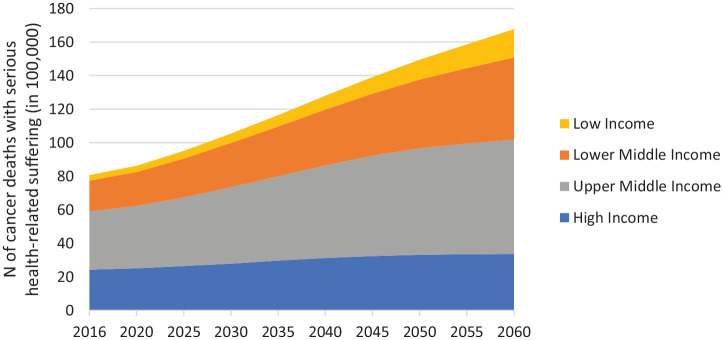
Projected increase in burden of serious health-related suffering associated with cancer, in World Bank income regions 2016 to 2060. *Number of deaths in 100,000s.*

By 2060, there will be an estimated 16.3 million people dying with cancer each year who experience serious health-related suffering (compared to 7.8 million in 2016), 80% of whom will be in low and middle income countries. The number of people who die from cancer and experience serious health-related suffering will increase in all world regions, but proportionately more in low income countries (407% increase 2016–2060 to 1.65 million people each year) compared to lower-middle income countries (169% increase 2016–2060 to 4.76 million people each year), upper-middle income countries (96% increase 2016–2060 to 6.67 million people each year), and high income countries (39% increase 2016–2060 to 3.23 million people each year) (Figure 1).

**Figure 2.**
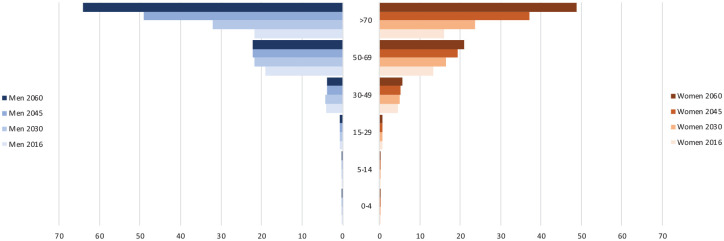
Changes in the number of people dying with serious health-related suffering associated with cancer, for global population stratified by age group and sex. *Number of deaths in 100,000s.*

Globally, the burden of serious health-related suffering among cancer decedents is projected to increase rapidly among people aged over 70 (Figure 2). By 2060, 67% of people who die with cancer and experience serious health-related suffering will be over 70 years old, compared to 47% in 2016. The proportion of people aged over 70 will be highest in high income countries, where, by 2060, 83% of people who die with cancer and experience serious health-related suffering will be over 70, compared with 71% in upper-middle income countries, 52% in lower-middle income countries, and 38% in low income countries. By 2060, 54% of people who die with cancer and experience serious health-related suffering will be men, and of those over 70 years 57% will be men.

**Figure 3.**
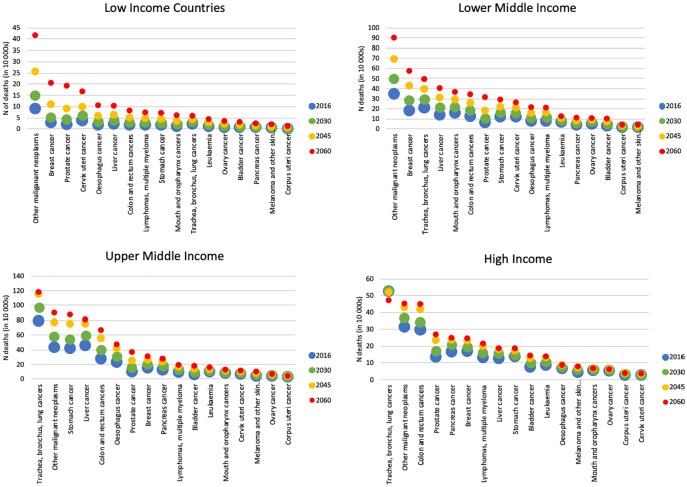
Number of deaths with serious health-related suffering according to cancer type, in low, lower-middle, upper-middle and high income countries. *Number of deaths in 10,000s.*

In high and upper-middle income countries, lung cancer will be the single greatest contributor to the burden of serious health-related suffering among cancer decedents in 2060, though the number of people in high income countries dying with lung cancer who experience serious health-related suffering is projected to fall between 2030 and 2060. In low and lower-middle income countries, breast cancer will be the single greatest contributor to serious health-related suffering among cancer decedents (Figure 3).

## Discussion

This study provides the first global projections of serious health-related suffering among cancer decedents by age, gender, cancer sub-type and world region. By 2060 an estimated 16.3 million people will die each year with cancer-associated serious health-related suffering, and needs are rising fastest in low income countries. Lung cancer (in high and upper-middle income countries) and breast cancer (in low and lower-middle income countries) will be the biggest contributors to serious health-related suffering among cancer decedents in 2060.

A consequence of the successes of global health initiatives in reducing mortality from infectious disease is the projected rise in patients requiring management of chronic progressive conditions such as cancer. Therefore, clinical, policy and research agendas must now refocus. Cancer prevention and treatment should be pursued alongside the joint priority of relief of patients’ suffering. This is relevant in all world regions, but is particularly important in low and middle income countries, where the projected increase in serious health-related suffering among cancer decedents is greatest and current access to palliative care is woefully inadequate.

By 2030 70% of global cancer cases will be in low and middle income countries,^9^ where late presentation and fewer resources lead to poor cure rates compared to high income countries.^10^ In India, a cancer diagnosis leads to catastrophic out-of-pocket expenditures that negatively affect both the patient and their family.^11^ Households affected by cancer report lower workforce participation and higher rates of borrowing and asset sales, compounding their poverty.^12^ Over a third of advanced cancer patients have information regarding their diagnosis withheld, and this is associated with poorer patient health-related quality of life.^13^ Within sub-Saharan Africa, patients with advanced cancer have a high burden of physical and psychological symptoms.^14^ Patients state that they would prefer open and honest communication around their needs and care options, yet perceive that this does not occur.^15^ Their family caregivers face financial hardship and psychological distress.^16^

There is increasing evidence that palliative care is effective and cost-effective in both high and low and middle income countries. For these reasons, the 2014 World Health Assembly (WHA) resolution called for all governments to integrate palliative care into their health plans.^17^ However, it is estimated that just 14% of the people who need palliative care receive it, and most of these people are in high income countries.^18^ Just 30/198 countries surveyed in 2017 had advanced integration of palliative care services, and development of palliative care services outside the ‘global North’ has been slow.^19^ Barriers to palliative care in low and middle income countries include lack of human resources, financial constraints, limited political commitment, restrictive pharmacovigilance regulation, challenges with drug importation processes and health system fragmentation.^20^

This study shares the limitations of other population-based approaches to assessment of palliative care needs, in that it relies on mortality data. The accuracy of mortality data is a particular issue in low and lower-middle income countries. We used the most up to date global mortality projections available, but unforeseen improvements and/or crises could affect the accuracy of these. We chose the method of the Lancet commission on palliative care and pain relief to estimate palliative care need, based on serious health-related suffering. This method aims to provide an indication of needs, rather than a precise number. In particular it does not take into account suffering associated with multimorbidity which may exacerbate symptom intensity. Our projections are therefore likely to underestimate true future need, particularly given the projected increase in proportion of people who die aged over 70. It is important to note that our estimates include only suffering among cancer decedents. The Lancet commission found that in 2015 7.6 million cancer decedents and 7.1 million non-decedents experienced serious health-related suffering. Therefore the future burden of suffering among decedents and non-decedents together could be around two-fold greater than our estimates.

Our projections warn that many people with cancer will die with unnecessary suffering unless there is expansion of cancer programmes that integrate palliative care. Failure to respond to this challenge will be damaging for both the individuals affected and the health systems within which they are treated.
